# 

**Published:** 1857-06

**Authors:** 


					﻿No. I.]
JUNE.
[Vol. XIII.
BUFFALO
MEDICAL JOURNAL
AND
jlttmtljh) lieu i civ
or
MEDICAL AND SURGICAL SCIENCE.
SANFORD B. HUNT, M. D.,
ZE Kia? OK.
BUFFALO, N. Y.:	.
E . ft. J E W E T T <fc CO.’S STEAM P li E S S.
Commercial Advertiser Buildings.
1857.
Price, $2.00 in advance, or $2.50 at the end of three months.
				

